# Impaired event-related theta spectral coherence in emotional facial expression processing in neurodegenerative disorders

**DOI:** 10.3389/fnhum.2026.1708832

**Published:** 2026-04-16

**Authors:** Hakan Uzunlar, Rümeysa Duygun, İlayda Kıyı-Atilla, Tuba Aktürk, Ebru Yıldırım, Nesrin Helvacı Yılmaz, Lütfü Hanoğlu, Henrique Sequeira, David Papo, Massimiliano Zanin, Claudio Babiloni, Görsev Yener, Bahar Güntekin

**Affiliations:** 1 Department of Neuroscience, Graduate School of Health Sciences, Istanbul Medipol University, Istanbul, Türkiye; 2 Research Institute for Health Sciences and Technologies (SABITA), Neuroscience Research Center, Istanbul Medipol University, Istanbul, Türkiye; 3 School of Psychology, University of Sussex, Brighton, United Kingdom; 4 Department of Neurosciences, Institute of Health Sciences, Dokuz Eylül University, Izmir, Türkiye; 5 Section Brain Stimulation and Cognition, Department of Cognitive Neuroscience, Faculty of Psychology and Neuroscience, Maastricht University, Maastricht, Netherlands; 6 Neuroscience and Neurotechnology Center of Excellence (NÖROM), Gazi University, Ankara, Türkiye; 7 Faculty of Medicine, Department of Neurology, Istanbul Medipol University, Istanbul, Türkiye; 8 University of Lille, CNRS, UMR 9193 - SCALab - Sciences Cognitives et Sciences Affectives, Lille, France; 9 Department of Neuroscience and Rehabilitation, Section of Physiology, University of Ferrara, Ferrara, Italy; 10 Center for Translational Neurophysiology, Fondazione Istituto Italiano di Tecnologia, Ferrara, Italy; 11 Instituto de Física Interdisciplinar y Sistemas Complejos IFISC (CSIC-UIB), Parc Bit, Palma de Mallorca, Spain; 12 Department of Physiology and Pharmacology “V. Erspamer”, Sapienza University of Rome, Rome, Italy; 13 IRCCS San Raffaele Roma - Cassino, Cassino Site, Cassino, Italy; 14 Department of Neurology, Medical School, Dokuz Eylul University, Izmir, Türkiye; 15 IBG: International Biomedicine and Genome Center, Izmir, Türkiye; 16 Department of Biophysics, School of Medicine, Istanbul Medipol University, Istanbul, Türkiye

**Keywords:** Alzheimer’s disease, EEG, emotional facial expressions, event-related theta spectral coherence, Parkinson’s disease

## Abstract

**Introduction:**

The ability to recognize emotional facial expressions relies on brain functional networks, particularly in the right hemisphere, and is impaired in neurodegenerative conditions such as Alzheimer’s disease and Parkinson’s disease. Given that this ability involves brain networks operating within hundreds of milliseconds, we tested the hypothesis of abnormalities in event-related spectral electroencephalographic coherence, with a focus on the right versus left hemispheres in these patients.

**Methods:**

Event-related theta (4–7 Hz) magnitude-squared coherence was calculated for both intra-hemispheric (frontal–temporal, frontal–parietal, central–temporal, and related pairs) and inter-hemispheric homologous electrode pairs (F3–F4, C3–C4, T7–T8, TP7–TP8, P3–P4, O1–O2). We enrolled 25 patients with amnestic mild cognitive impairment, 15 Parkinson’s disease patients with mild cognitive impairment, 25 Alzheimer’s disease patients with dementia, and 16 Parkinson’s disease patients with dementia, along with 25 healthy elderly as controls. Participants were presented with three different facial expressions (angry, happy, and neutral) 60 times each in a pseudo-random order.

**Results:**

Theta (4–7 Hz) spectral coherence was higher in the right than the left hemisphere across all groups, with Parkinson’s disease patients exhibiting the lowest values. Moreover, interhemispheric theta coherence was lower in Parkinson’s disease patients with mild cognitive impairment and dementia compared to the amnestic mild cognitive impairment and healthy elderly groups.

**Discussion:**

These findings indicate that cortical functional connectivity related to emotional facial expression processing is more disrupted in Parkinson’s disease than in Alzheimer’s disease, at both mild cognitive impairment and dementia stages. This disruption affects not only the right hemisphere but also interhemispheric connectivity. Finally, it would occur at theta frequencies.

## 1 Introduction

The ability to recognize emotions is essential in our daily lives. It regulates social behavior (Seidel et al., 2010) and aids in survival (Pritsch et al., 2017). The most important way of conveying emotions is through facial expressions. So, the integrity of facial emotion recognition is critical to maintain social functioning. Unfortunately, like other aspects of sensory and cognitive processes, the ability to process emotional facial expressions is affected by physiological (Ruffman et al., 2008; Gonçalves et al., 2018) and pathological (Bora et al., 2016) aging. In line with this, numerous studies have demonstrated a decline in emotional facial expression recognition in patients with neurodegenerative conditions such as Alzheimer’s disease (AD) (Spoletini et al., 2008) and Parkinson’s disease (PD) (Ricciardi et al., 2017; Chiang et al., 2024). The recognition of facial expressions was impaired in patients with mild cognitive impairment (MCI) (Bora and Yener, 2017), frontotemporal dementia (Lavenu et al., 1999; Rosen et al., 2006), and AD with dementia (ADD). Among these patient groups, the investigation of the recognition of facial expressions has mostly been performed with AD patients. A large number of these studies showed that there was a deficit in facial expression recognition in AD patients (McLellan et al., 2008). Furthermore, sub-cortical structures impacted by PD pathology are known to play a role in emotion processing (Moonen et al., 2017). As a corollary, impairments in recognition of emotional facial expression performance are commonly observed in PD patients, especially with recognizing negative emotions (Gray and Tickle-Degnen, 2010; Baggio et al., 2012; Argaud et al., 2018; Siquier and Andrés, 2022).

The recognition of emotional facial expressions depends on brain functional networks, particularly within the right hemisphere (Palomero-Gallagher and Amunts, 2022). This view is supported by prominent hypotheses: the right hemisphere hypothesis (Borod et al., 1998), which suggests right-hemispheric dominance for all emotions, and the valence hypothesis (Davidson, 1992), which associates the right hemisphere with the processing of negatively valenced emotions. Given that this ability involves brain networks operating within hundreds of milliseconds, electroencephalographic (EEG) techniques were used to explore abnormalities in PD and AD patients (Ricciardi et al., 2017; McLellan et al., 2008; Hot et al., 2013). Local abnormalities in EEG rhythms were measured by spectral power density (Jaramillo-Jimenez et al., 2023). In contrast, abnormal interdependence of EEG rhythms at electrode or source pairs was computed by spectral coherence or related procedures (De Hemptinne et al., 2013). In event-related oscillations (ERO) studies, beta (13–30 Hz) and gamma (>30 Hz) responses were associated with angry and fearful facial expressions (Güntekin and Başar, 2007; Jung et al., 2011; Luo et al., 2007; Sato et al., 2011). In contrast, positive and negative emotional facial expressions induced higher ERO delta and theta responses compared to neutral faces (Balconi and Lucchiari, 2006; Güntekin and Başar, 2014; Knyazev et al., 2009). Posterior ERO delta (Güntekin and Başar, 2014; Knyazev et al., 2009) and theta (Güntekin and Başar, 2007) responses were also associated with emotional facial expressions. Along this research line, patients with AD dementia (ADD) (Güntekin et al., 2019) and those with PD dementia (PDD) (Yuvaraj et al., 2015; Yuvaraj et al., 2016) showed lower event-related EEG theta power and coherence values in response to emotional facial expressions compared to matched cognitively unimpaired healthy control participants.

Although this is still being debated, it has been suggested that AD (Lavenu et al., 1999; Hargrave et al., 2002; Heilman and Nadeau, 2022) and PD (Mattavelli et al., 2021) patients may have a selective impairment in labeling negative emotions (e.g., fear, contempt, sadness, disgust). The limbic (Ávila-Villanueva et al., 2022) and basal ganglia structures (Liu et al., 2020) are known to be particularly affected by these pathologies, and amygdala damage is seen in both of these disorders (Nikolenko et al., 2020). Specifically, pathophysiological processes in AD are known to involve atrophy that is primarily affecting frontal and temporal regions, and posterior cortical areas in the earlier stages of the disease (Harper et al., 2017). Moreover, the loss of cholinergic neurons is one of the most significant events. Parkinson’s disease pathophysiology, on the other hand, known to lead to the loss of dopaminergic neurons in the midbrain, particularly in the substantia nigra, and cause motor symptoms (MacMahon Copas et al., 2021). As a result, a comparison between patients with these diseases is likely to yield significant topographical group differences in their event-related EEG brain activity. Furthermore, cortical changes in these structures may contribute to alterations in large-scale functional connectivity associated with emotional processing, especially in terms of negative emotions.

The present study aimed to investigate alterations in event-related EEG theta spectral coherence during emotional facial expression processing in individuals with different neurocognitive disorders. Given the role of large-scale functional networks in rapid emotional processing, we examined intra- and inter-hemispheric coherence patterns in Alzheimer’s disease (AD) and Parkinson’s disease (PD) at both mild cognitive impairment (MCI) and dementia stages. By comparing these groups with healthy elderly controls within the same experimental paradigm, the study sought to explore whether disease type and cognitive severity are associated with differences in functional connectivity patterns. In addition, considering theoretical models emphasizing right-hemispheric involvement in emotional processing and prior evidence suggesting sensitivity to negative emotional stimuli, we examined hemispheric asymmetry and the potential modulation of theta coherence by emotional valence (angry, happy, and neutral facial expressions).

## 2 Methods

### 2.1 Participants

The sample of this study consisted of 106 participants, including 25 HE, 25 aMCI, 15 PD-MCI, 25 ADD, and 16 PDD patients. The differences in age between groups were not statistically significant. Education years, gender, and MMSE scores differed between groups. The demographic and clinical information for each group is displayed in detail in Table 1.

**Table 1:** Demographic information of all participants.

Participants	Gender*	Age	Education*	MMSE*
HE	11F/14M	70.44 ± 6.8	10.72 ± 5.2	28.64 ± 1.6
aMCI	13F/12M	71.48 ± 5.1	8.32 ± 5.4	25.32 ± 1.8
PD-MCI	5F/10M	71.66 ± 6.4	1.93 ± 5.2	26.00 ± 2.8
ADD	18F/7M	71.84 ± 7.2	8.36 ± 4.6	19.32 ± 4.6
PDD	3F/13M	72.43 ± 6.8	5.68 ± 5.4	19.37 ± 3.8

“*” is used to indicate the presence of significant differences among the groups (*p* < 0.05).

All participants underwent neurological examination and neuropsychological testing to aid in differential diagnosis. For general cognitive screening, the Turkish version of the Standardized Mini Mental Test (MMSE) (Güngen et al., 2002) was used. Memory functions were evaluated using the Verbal Memory Processes Test (SBST) (Öktem Tanör, 2011) and the Wechsler Memory Scale-Revised (WMS-R) visual reproduction subtest (Wechsler, 1987). Attention was assessed with the WMS-R digit span test, and executive functions with the Stroop Color Word Test (Karakaş, 2006), clock drawing test (Brodaty and Moore, 1997), WMS-R similarities test, verbal fluency tests (Crawford et al., 1992), and the Trail Making Test (Reitan, 1958). Language functions were assessed using the Boston Naming Test (Kaplan et al., 2001). Visuospatial abilities were evaluated with simple picture copying, Benton Face Recognition, and the Turkish version of the Line Orientation Test (BLOT) (Karakaş, 2006). In addition, the clinical status evaluation included the Clinical Dementia Rating Scale (CDR) (Morris, 1993), Geriatric Depression Scale (GDS) (Sheikh and Yesavage, 1986), and Neuropsychiatric Inventory (NPI) (Cummings, 1997). However, within the scope of this paper, these scales were used to establish eligibility, and only the MMSE scores were analyzed.

**Figure 1:**
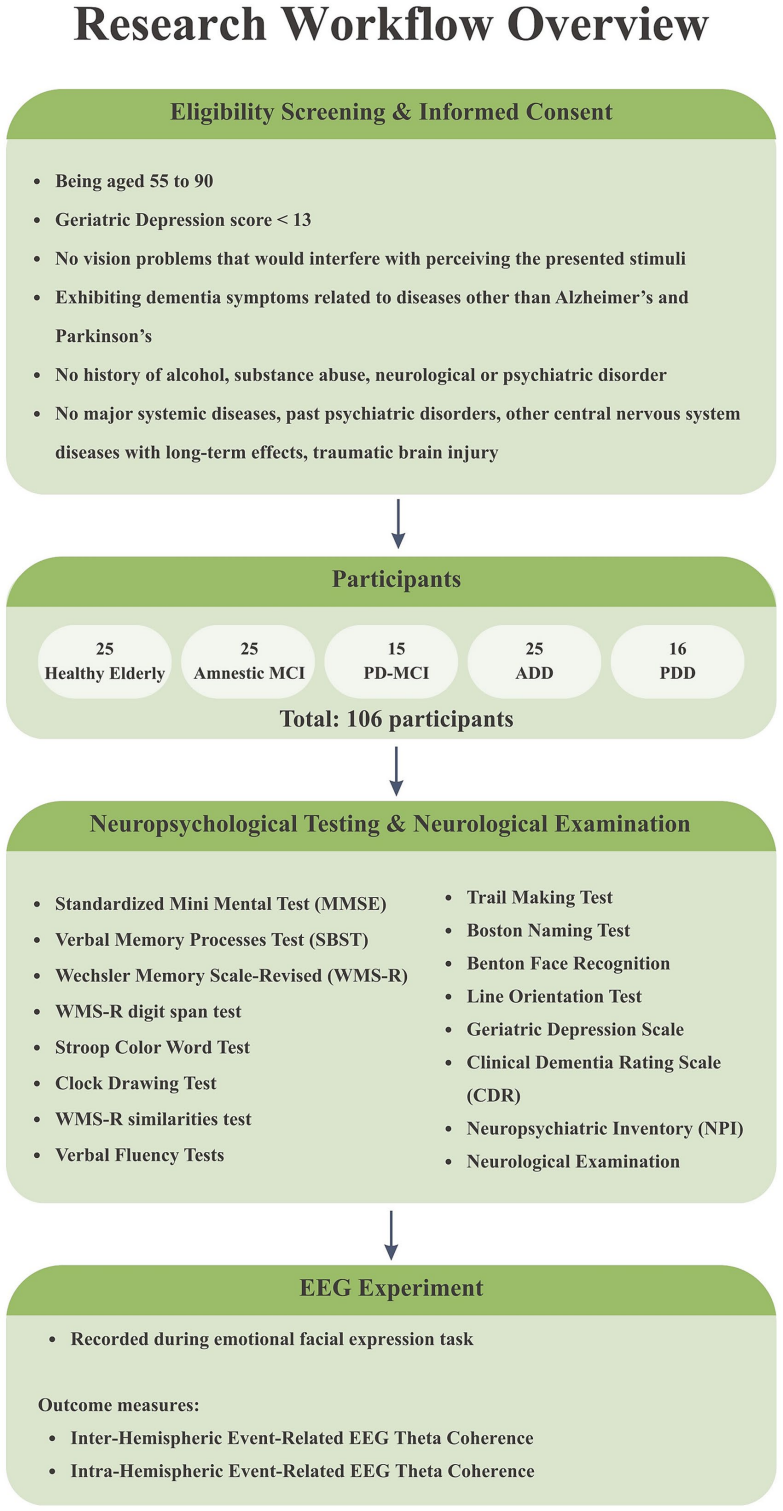
Study workflow showing participant selection, participant distribution across diagnostic groups, neuropsychological testing, and the experimental task.

Inclusion criteria for all groups included voluntary participation, being aged 55 to 90, and having a Geriatric Depression score of less than 13. The exclusion criteria included failing to sign the informed consent form, withdrawing from the study, having vision problems that would interfere with perceiving the presented stimuli, exhibiting dementia symptoms related to diseases other than Alzheimer’s and Parkinson’s, and a history of alcohol or substance abuse. Additionally, participants with major systemic diseases, past psychiatric disorders such as major depression, epilepsy, or a history of stroke or other central nervous system diseases with long-term effects, as well as those with traumatic brain injury, were excluded from the study. The study workflow is additionally summarized in a flowchart (see Figure 1) for clarity.

The following inclusion and exclusion criteria were defined specifically for each group:

#### 2.1.1 Healthy elderly

Participants in the HE group had no history of neurological, psychiatric, or systemic disease, were not taking acetylcholinesterase inhibitors or any psychoactive drugs that affect cognitive functions, did not have general cognitive impairment (SMMT score ≥ 27), did not meet any diagnostic criteria as a result of their neuropsychological evaluation, and had no neurological abnormality detected in their neurological examination.

#### 2.1.2 Amnestic mild cognitive impairment

The aMCI Individuals had scores that objectively deviated 1.5 standard deviations from the mean in the negative direction on a battery of neuropsychological tests. They were classified as aMCI based on whether they had primarily memory problems or problems affecting multiple cognitive domains. They also had CDR scores of 0.5 and were maintaining their individual and social functionality.

#### 2.1.3 Parkinson’s disease with mild cognitive impairment

For the PD-MCI, we included participants who were diagnosed according to the Movement Disorders Society’s diagnostic criteria and via the Unified Parkinson’s Disease Rating Scale (UPDRS) (Martínez-Martín et al., 1994). The criteria regarding their neuropsychological test scores were the same as those mentioned above for the amnestic mild cognitive impairment group. In addition, they were maintaining their individual and social functionality.

#### 2.1.4 Alzheimer’s dementia

The ADD patients were included based on receiving ADD diagnosis according to the diagnostic criteria of the National Institute on Aging and the Alzheimer’s Association (NIA-AA) in the United States.

#### 2.1.5 Parkinson’s dementia

Participants were assigned to the PDD group if they were diagnosed with Parkinson’s disease using the Movement Disorders Society’s diagnostic criteria and the Unified Parkinson’s Disease Rating Scale (UPDRS) and had scores that objectively deviating 1.5 standard deviations from the mean at least one (or more) cognitive domain, as well as a loss of individual and social functioning.

**Figure 2:**
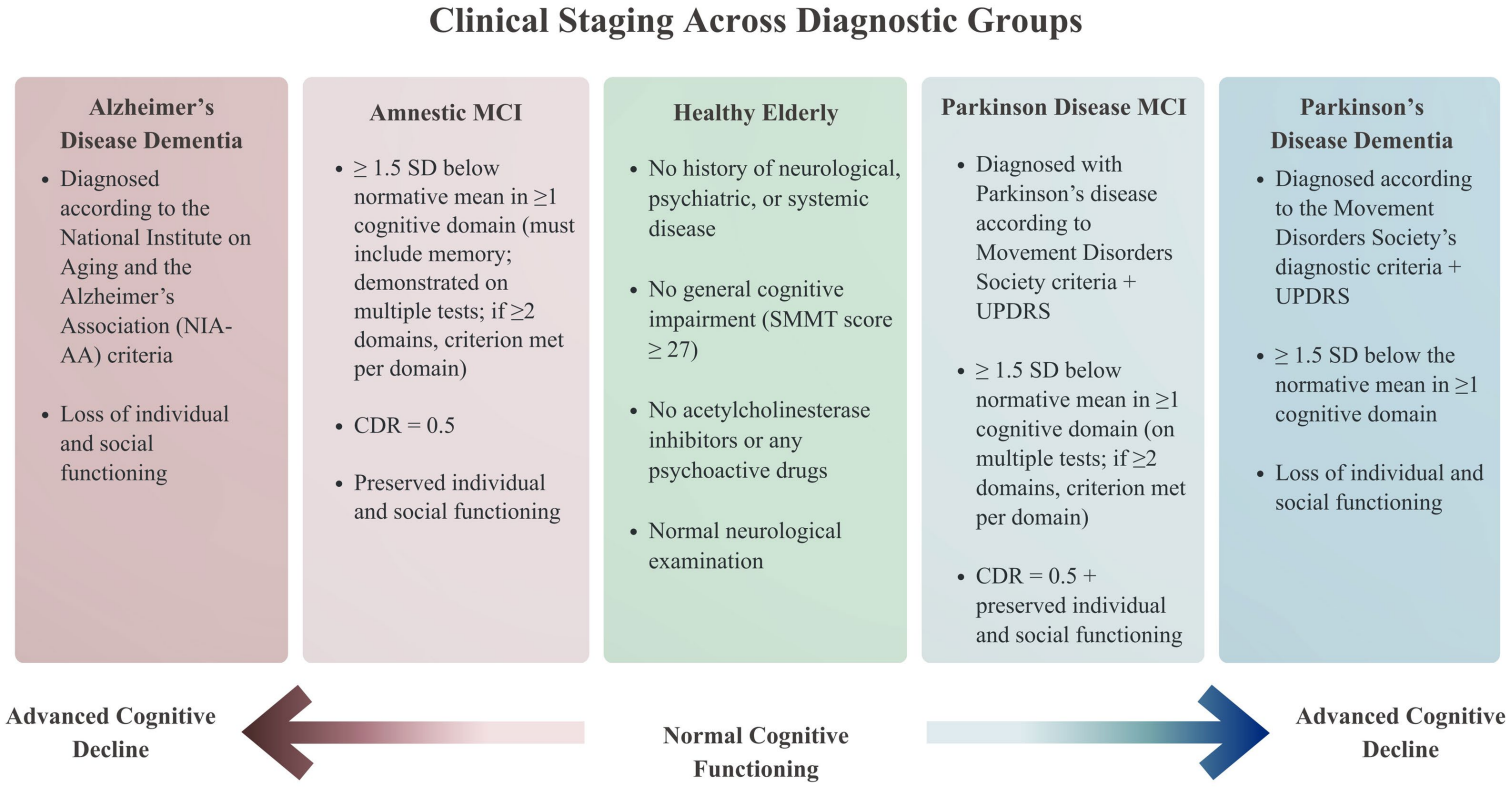
Clinical staging across diagnostic groups and their defining criteria.

An overview of the classification of disease severity is depicted in Figure 2.

This study was approved by the Istanbul Medipol University Non-invasive Clinical Research Ethics Committee (Approval No: 801, Date: 22/10/2020). All procedures were conducted in accordance with the Declaration of Helsinki. Written informed consent was obtained from all participants prior to participation.

### 2.2 Experimental design and procedure

All participants underwent a neuropsychological evaluation following their neurological examination at the clinic. They were then prepped for the EEG recording and sat across the screen (19″, 5:4) in an isolated room. The presentation of the emotional facial expressions paradigm then followed this. A set of trial stimuli preceded each facial expression segment in the paradigm. The paradigm included three different women’s facial expressions (angry, happy and neutral). At the end of each facial expression condition, we had participants rate the valence and arousal of each stimulus on the Self-Assessment Mannequin (SAM) from 0 to 9 points.

### 2.3 Stimuli

Emotional facial expression stimuli used in this study were selected from Ekman and Friesen’s (1976) Pictures of Facial Affect collection, which are standardized pictures commonly used in research. Each facial expression was presented 60 times in a pseudo-random order for each paradigm with a 3–7 s inter-stimulus interval, and it stayed on the screen for 1 s. The paradigm was designed using a MATLAB code.

### 2.4 EEG recording

BrainVision 32-channel DC amplifier hardware and BrainVision Recorder software were used to record EEG data (Brain Products, Munich, Germany). The same company’s elastic caps (Easy-cap) were used for electrode placement on the scalp in accordance with the international 10–20 system. They had 30 scalp electrodes (Fp1, Fp1, F7, F3, Fz, F4, F8, FT7, FC3, FCz, FC4, FT8, T7, C3, Cz, C4, T8, TP7, CP3, CPz, CP4, TP8, P7, P3, Pz, P4, P8, O1, Oz, O2) and two ocular electrodes (HEOG, VEOG), as well as two reference electrodes placed on both earlobes (A1 + A2). Data were collected at a 500 Hz sampling rate and 0.01 Hz - 250 Hz band limit. Impedance values for the reference and ground electrodes were kept under 5 kΩ, and the remaining electrodes were kept under 10 kΩ. Recordings took place in a dimly lit room that was isolated from sound and electromagnetic waves. The subjects sat 100 cm away from the screen where the stimuli were displayed. Throughout the recording session, participants were monitored via a night vision camera and communicated with a diaphone.

### 2.5 EEG data analysis

All EEG data were analyzed using the standardized preprocessing steps via BrainVision Analyzer 2. Firstly, a bandpass filter in the 0.01–60 Hz range and a notch filter at 50 Hz were applied. Then, blinking and other eye-movement-related components were removed using Independent Component Analysis (ICA). Data were then segmented into 2,000 ms epochs covering the time window of 1,000 ms before and 1,000 ms after the stimulus onset. Finally, epochs with motor and instrumental artifacts were removed. Current Source Density (Order of Splines: 4, Maximal Degree of Legendre Polynomials: 10, Default Lambda: 1E-05) was applied to all epochs after the segmentation, to remove the reference values from the data and reduce the effects of volume conduction. Continuous Wavelet Transform was used to calculate the cross-spectrum of each epoch. This was performed on 2-s epochs, in the 3–8 Hz frequency range, with “cycle” and “frequency steps” parameters set to “3” and “10,” respectively, with the logarithmic steps option selected. We preferred Gabor normalization. Additionally, complex values of wavelet coefficients were preserved for use in coherence analysis. In the stage of calculating the cross-frequency values, the data in the time domain were transferred to the time-frequency domain.

Coherence was calculated using cross-spectrum values via tools on BrainVision Analyzer 2. The preferred method was magnitude squared coherence (MSC). Coherence values were calculated using values from electrode pairs at frontal (F3-F4), temporal (T7-T8), temporo-parietal (TP7-TP8), central (C3-C4), parietal (P3-P4), and occipital (O1-O2) areas. These electrode pairs were specifically selected due to displaying significant facial expressions-related changes and being associated with neural losses in various neurodegenerative disorders (Güntekin et al., 2019; Yuvaraj et al., 2015). Besides, to investigate connectivity between distant and adjacent locations, frontal (F3/4–P3/4, F3/4–T7/8, F3/4–TP7/8, F3/4–O1/2) and central (C3/4–T7/8, C3/4–Tp7/8, C3/4–O1/2) electrode pairs were used, respectively. Although the prominent measurement of connectivity in this study was magnitude-squared coherence, the imaginary part of coherence (IPC) was also calculated to eliminate the possible effects of volume conduction. With this calculation, we aimed to compare the coherence coefficients and the imaginary part of coherence and detect values that indicated unrealistic connectivity. Furthermore, to normalize our data, we performed Fisher’s *Z*-transform and converted the Pearson correlation coefficients to standardized *Z* scores. These values were then used in further statistical analyses.

### 2.6 Statistical analysis

Variables such as age, gender, education, and MMSE scores were compared between groups using one-way ANOVA. Fisher’s *Z*-transformed coherence values were compared using repeated measures ANOVA. For all statistical tests, Greenhouse–Geisser corrected significance values (*p*) that were below 0.05 (*p* < 0.05) were accepted. The repeated measures ANOVA models in this study had one between-subjects variable (Groups: HE, aMCI, PD-MCI, ADD, and PDD) and 3 within-subjects variables: facial expressions (angry, happy, and neutral), locations and hemispheres.

In total, six repeated measures ANOVAs were conducted. Specifically, two of them were conducted for intra-hemispheric and inter-hemispheric connectivity separately. In the first model (3 Facial Expressions X 7 Locations X 2 Hemispheres X 5 Groups), which was for the intra-hemispheric connectivity, 7 electrode pairs were included representing the 7 locations on the right and left hemispheres (F3-T7 and F4-T8, F3-TP7 and F4-TP4, F3-P3 and F4-P4, F3-O1 and F4-O2, C3-T7 and C4-T8, C3-TP7 and C4-TP8, C3-O1 and C4-O2). The second repeated measures ANOVA was carried out as a follow-up analysis to more thoroughly examine hemispheric asymmetry in intra-hemispheric theta coherence, with Facial Expression, Hemisphere, and Group as factors. Mean coherence values were averaged across electrode pairs within each hemisphere (3 Facial Expression X 2 Hemispheres X 5 Groups). The third ANOVA model had a 3 (angry, happy, neutral) X 6 (F3-F4, C3-C4, T7-T8, TP7-TP8, P3-P4, O1-O2) X 5 (HE, aMCI, PD-MCI, ADD, PDD) design, which was conducted for the inter-hemispheric event-related EEG theta spectral coherence values. Finally, three condition-specific ANOVAs for each facial expression (angry, happy, neutral) were performed to further explore regional and group-level effects. Each model included Location (F3-F4, C3-C4, T7-T8, TP7-TP8, P3-P4, O1-O2) as a within-subjects factor and Group (HE, aMCI, PD-MCI, ADD, PDD) as a between-subjects factor (6 × 5 design). To address potential bias due to gender imbalance, particularly in the PD-MCI, ADD and PDD groups, and the exclusive use of female facial expression stimuli, Gender was included as an exploratory between-subjects factor in all six ANOVA models. As this was not part of the primary analysis plan, only the main effects of Gender and Gender × Group interactions are reported. All statistical analyses were performed using Jamovi 2.3.28 Software (The Jamovi Project, 2025).

## 3 Results

### 3.1 Intra-hemispheric event-related EEG theta coherence

To examine the differences in event-related EEG theta coherence among patient groups across different emotional facial expressions, a repeated measures ANOVA was conducted. A 3 (Facial Expressions: angry, happy and neutral) X 7 (Locations: F3-T7 and F4-T8, F3-TP7 and F4-TP8, F3-P3 and F4-P4, F3-O1 and F4-O2, C3-T7 and C4-T8, C3-TP7 and C4-TP8, C3-O1, and C4-O2) X 2 (Hemispheres: left and right) X 5 (Groups: HE, aMCI, PD-MCI, ADD, PDD) repeated measures ANOVA was performed to assess intra-hemispheric event-related EEG theta coherence. Although variability was observed, the main effect of group did not reach statistical significance (F (df = 4, 101) = 2.027, *p* = 0.096, *ηp*^2^ = 0.074), showing that the expected group-related differences in intra-hemispheric event-related EEG theta coherence were not supported. A significant interaction between group and location was observed. However, none of these differences remained statistically significant following Bonferroni correction and should therefore be interpreted with caution (See Table 2).

**Table 2:** All intra- and inter-hemispheric comparisons and significance levels.

Comparison	Intra-hemispheric 3 Face × 7 Loc × 2 Hem × 5 Group	Inter-hemispheric 3 Face × 6 Loc × 5 Group
	Group comparisons and interaction details (post-hoc)	
Group	Not significant	HE > PD-MCI**, PDD** HE > ADD aMCI > PD-MCI**, ADD*, PDD***
Group × Location	c–t: HE > PD-MCI, ADD, PDD c–tp: HE > PD-MCI, ADD f–o: aMCI > PD-MCI, PDD	HE >ADD: t, tp HE > PD-MCI: c, t, tp, o HE> PDD c, tp, o
Face*	Angry > Neutral*	Angry > Neutral**, Happy
Group × Face**	Not significant	Angry: HE > PD-MCI, ADD, PDD* Angry: aMCI > PD-MCI*, ADD*, PDD**
Hemisphere***	Right > Left***	–
Location***	f-p < c-tp, c-o (All *) f-tp > f-p, f-o (All *) f–o < f-t, c–t, c–tp, c–o (All ***)	o > f, c, p (All ***) tp > f, c, p (All ***) t > f, c, p (All ***)
Group × Face × Location***	Not significant	–

(*p* OR *p*_bonferroni:_ *<0.05, **<0.01, ***<0.001), HE: Healthy Elderly, aMCI: Amnestic Mild Cognitive Impairment, PD-MCI: Parkinson’s Disease-Mild Cognitive Impairment, ADD: Alzheimer’s Disease Dementia, PDD: Parkinson’s Disease Dementia, f: frontal, c: central, t: temporal, tp: temporoparietal, p: parietal, o: occipital, f-o: frontal-occipital, c-t: central-temporal, c-tp: central-temporoparietal, f-p: frontal–parietal, f-t: frontal-temporal, c-o: central-occipital.

**Figure 3:**
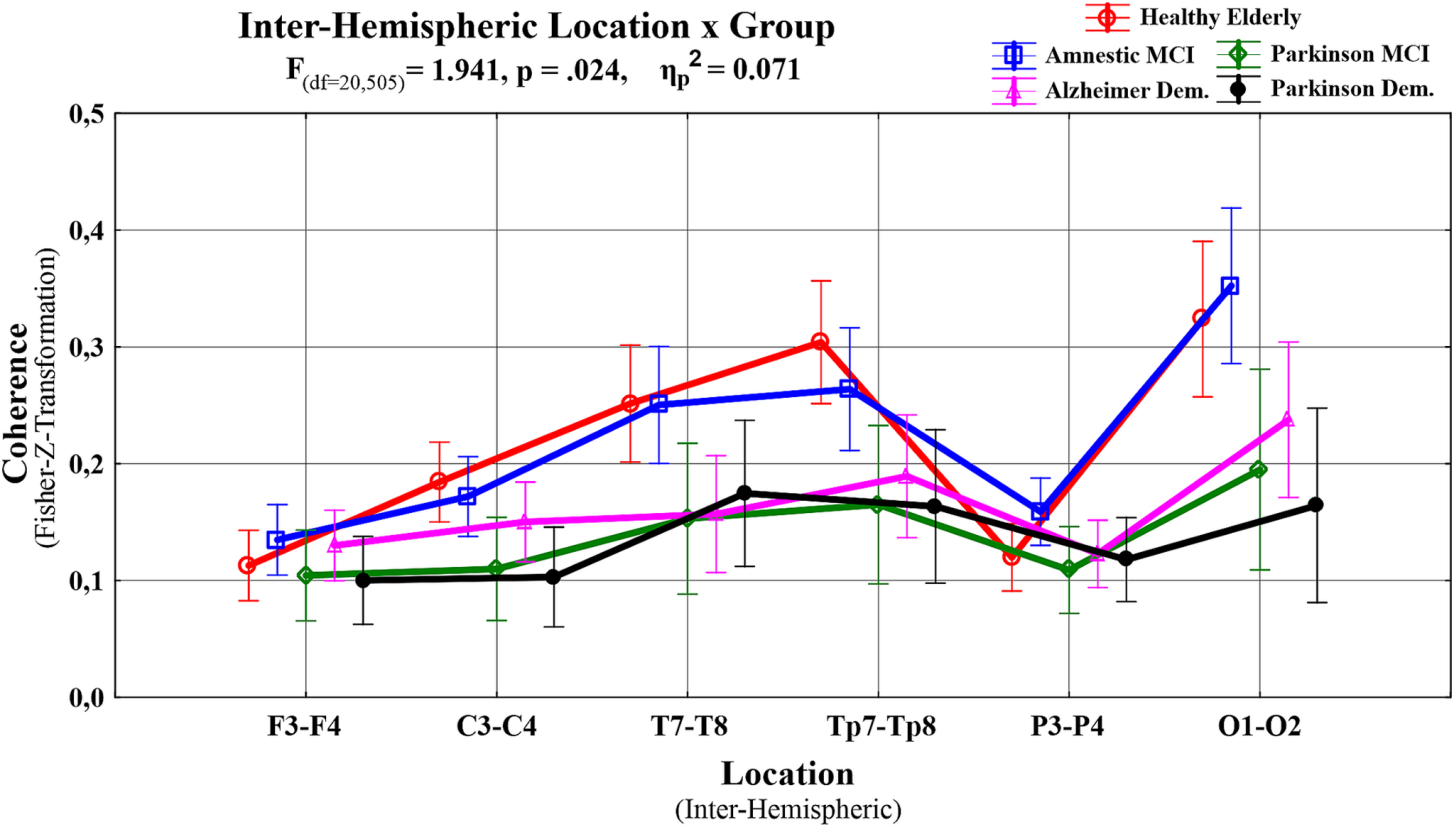
Event-related EEG theta (3–7.5 Hz) coherence between C3-T7 and C4-T8 electrode pairs in response to angry facial expressions; **(A)** Depicts the grand average visuals for HE, **(B)** for aMCI, **(C)** for PD-MCI, **(D)** for PDD, and **(E)** for ADD; the left and right columns represent coherence values for the left and right hemispheres, respectively, and each graph depicts grand averages from 1,000 ms before to 1,000 ms after stimulus presentation.

Beyond the primary hypothesis-driven results, additional significant main effects and interactions were identified. Firstly, the analysis revealed a significant main effect of Facial Expressions (*F*(df = 2,202) = 3.322, *p* = 0.038, *ηp*^2^ = 0.032). To further explore this effect, *post hoc* comparisons were conducted. These comparisons revealed that event-related EEG theta coherence values elicited by angry facial expressions were significantly higher than those elicited by neutral expressions (M = 0.0097, SE = 0.0038; *t*_101_ = 2.5309, *p* = 0.013, *p*_bonferroni_ = 0.039). Figure 3 illustrates intrahemispheric event-related EEG theta coherence between the C3–T7 and C4–T8 electrode pairs in response to angry facial expressions across groups. In addition, a significant main effect of Hemisphere (*F*(df = 1,101) = 13.521, *p* = 0.000, *ηp*^2^ = 0.118) was observed, indicating that intra-hemispheric event-related EEG theta coherence was higher in the right hemisphere compared to the left (M = −0.0140, SE = 0.0038; *t*_101_ = −3.6771, *p* < 0.001, *p*_bonferroni_ < 0.001). A significant main effect of Location was also observed (*F*(df = 6,606) = 9.287, *p* = 0.000, *ηp*^2^ = 0.084), reflecting variation in the intrahemispheric event-related EEG theta coherence values across recording sites. *Post hoc* comparisons revealed that theta coherence at frontal–parietal electrode pairs was significantly lower compared to central-temporoparietal (M = −0.0278, SE = 0.0075; *t*_101_ = −3.6873, *p* < 0.001, *p*_bonferroni_ = 0.008) and central-occipital sites (M = −0.0230, SE = 0.0073; *t*_101_ = −3.1642, *p* = 0.002, *p*_bonferroni_ = 0.043). In addition, coherence values at frontal–parietal (M = 0.0135, SE = 0.0039; *t*_101_ = 3.4558, *p* < 0.001, *p*_bonferroni_ = 0.017) and frontal-occipital electrode pairs (M = 0.0232, SE = 0.0042; *t*_101_ = 5.5209, *p* < 0.001, *p*_bonferroni_ < 0.001) were lower than frontal-temporoparietal electrode pairs (M = 0.1068, SE = 0.0046). Frontal–occipital coherence values were also significantly lower than those at frontal-temporal (M = 0.0179, SE = 0.0038; *t*_101_ = 4.7541, *p* < 0.001, *p*_bonferroni_ < 0.001), central–temporal (M = −0.0333, SE = 0.0073; *t*_101_ = −4.5694, *p* < 0.001, *p*_bonferroni_ < 0.001), central–temporoparietal (M = −0.0375, SE = 0.0070; *t*_101_ = −5.3227, *p* < 0.001, *p*_bonferroni_ < 0.001) and central–occipital electrode pairs (M = −0.0327, SE = 0.0067; *t*_101_ = −4.8668, *p* < 0.001, *p*_bonferroni_ < 0.001). All comparison results are shown in Table 2.

To more directly assess whether hemispheric asymmetry in theta coherence varies as a function of emotional facial expression and patient group, a simplified follow-up ANOVA was conducted with Facial Expression (angry, happy, neutral), Hemisphere (left, right), and Group (HE, aMCI, PD-MCI, ADD, PDD) as factors. In this model, mean coherence values were averaged across all left and right hemisphere electrode pairs. The analysis revealed that the three-way interaction among facial expression, hemisphere, and group did not reach statistical significance (*F*(df = 7, 191) = 1.0646, *p* = 0.389, *ηp*^2^ = 0.0405).

### 3.2 Inter-hemispheric event-related EEG theta coherence

**Figure 4:**
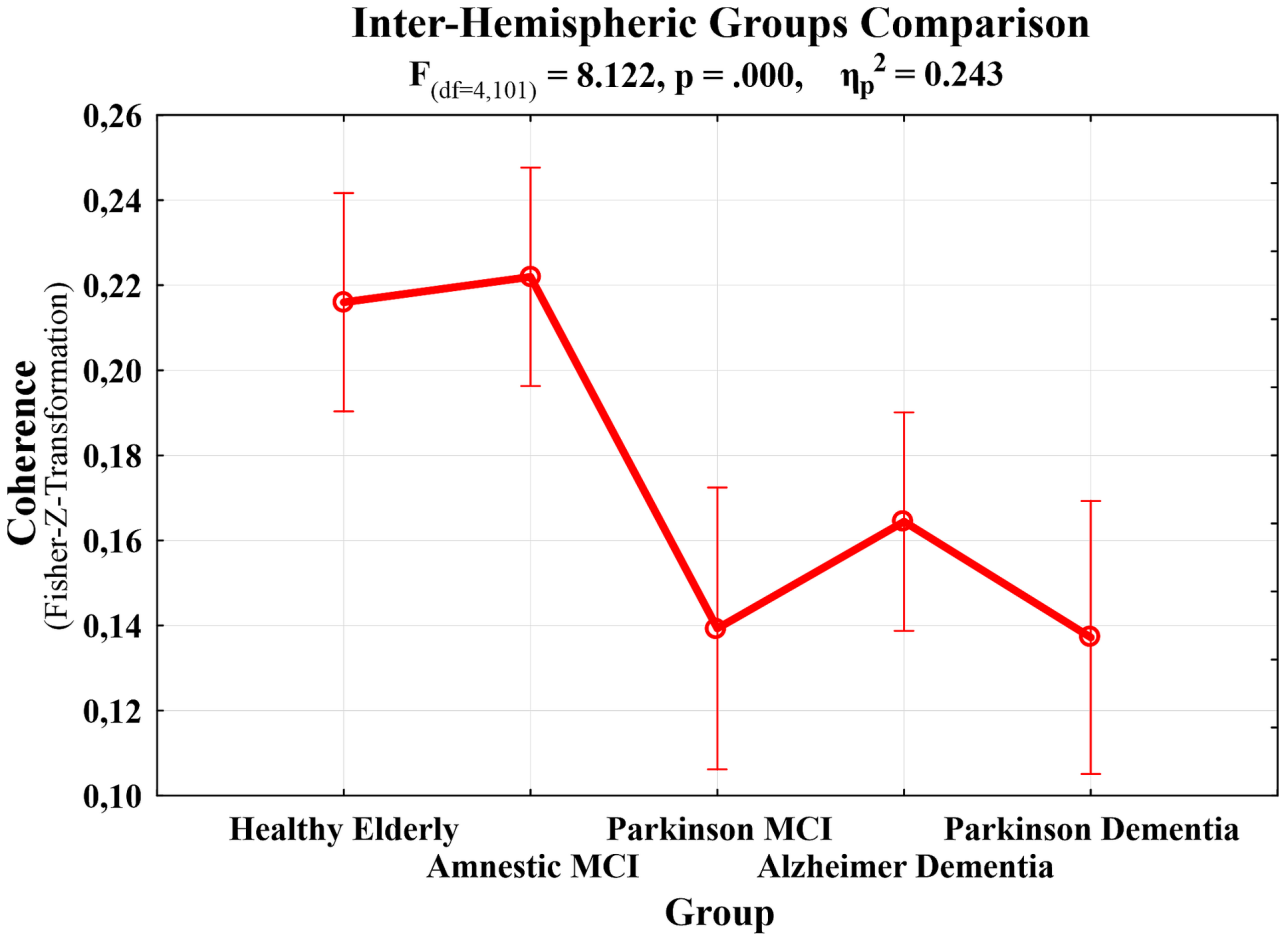
A line graph displaying the differences in terms of the Fisher’s *Z*-transformed inter-hemispheric event-related EEG theta coherence values among the HE (M = 0.2160, SE = 0.0129), aMCI (M = 0.2220, SE = 0.0129), PD-MCI (M = 0.1393, SE = 0.0167), ADD (M = 0.1644, SE = 0.0129), and PDD groups (M = 0.1372, SE = 0.0162).

**Figure 5:**
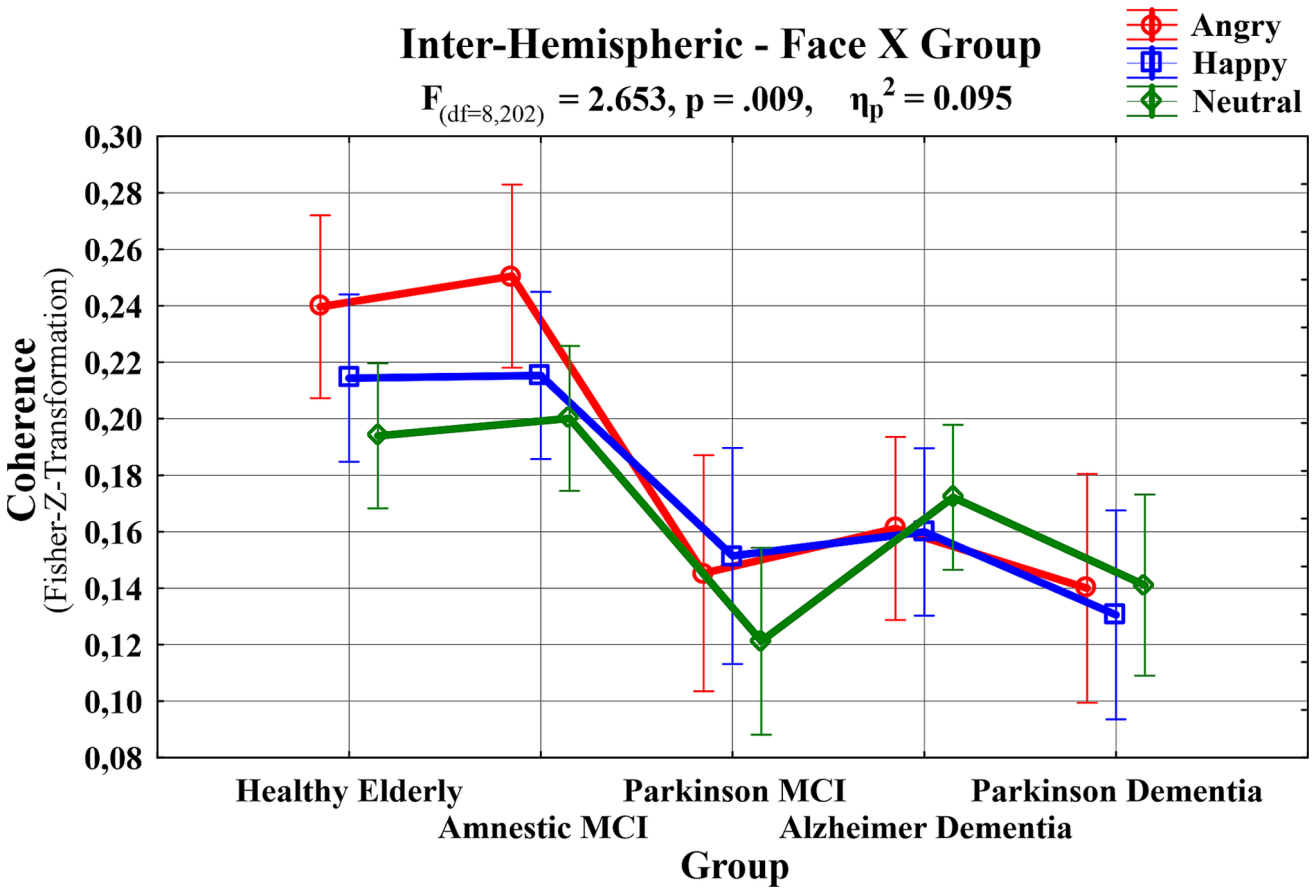
A line graph showing Fisher’s *Z*-transformed inter-hemispheric coherence values across groups (*F*(df = 8, 202) = 2.653, *p* = 0.009, *ηp*^2^ = 0.095); red line (circle) represents angry faces, blue line (square) represents happy faces, and green line (rhombus) represents neutral faces.

**Figure 6:**
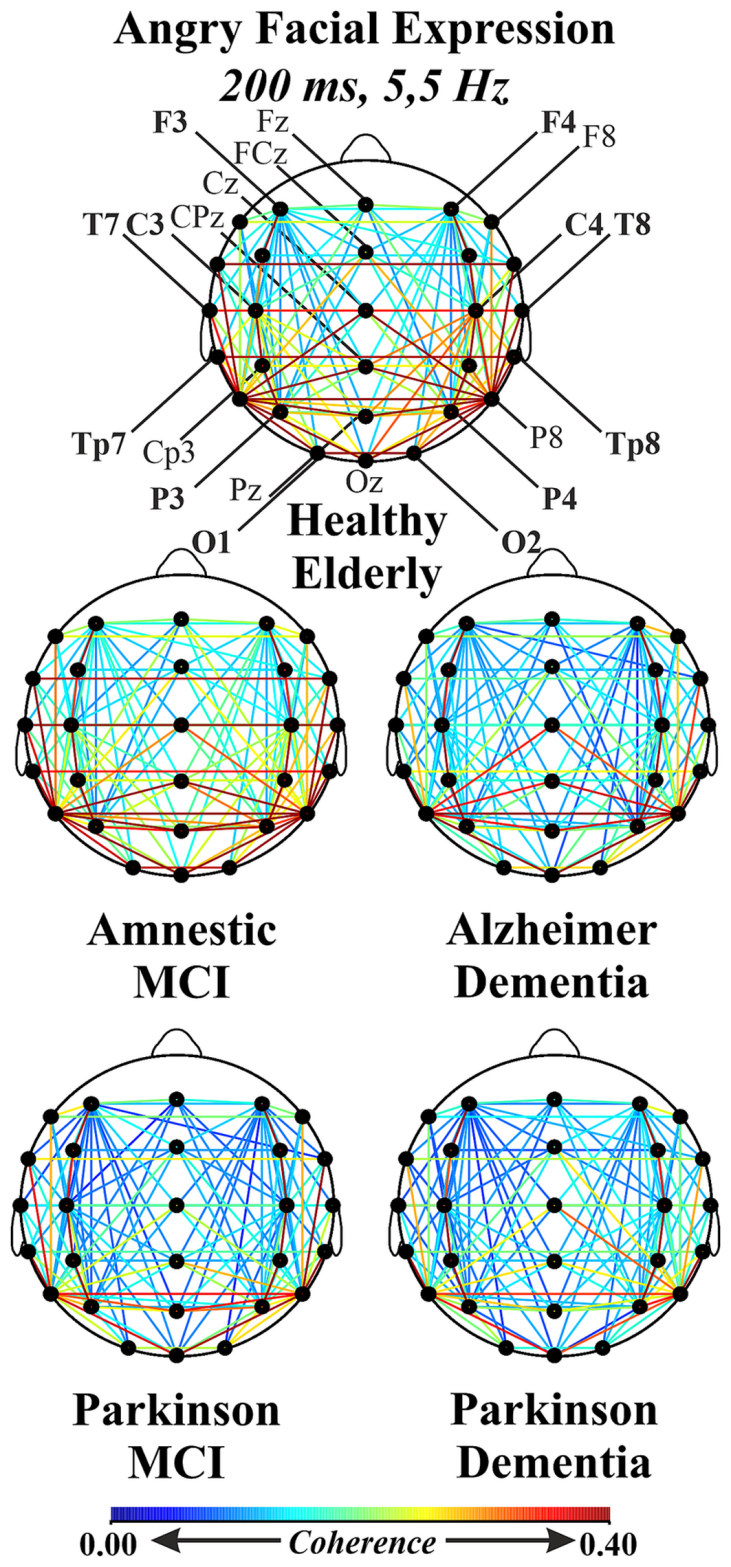
Group comparison (HE, aMCI, PD-MCI, ADD, and PDD) of inter-hemispheric event-related EEG theta coherence values at the occipital (O1–O2) electrode pairs: **(A)** Time-frequency representations of event-related EEG theta coherence values elicited by angry faces; **(B)** By happy faces; **(C)** By neutral faces.

**Figure 7:**
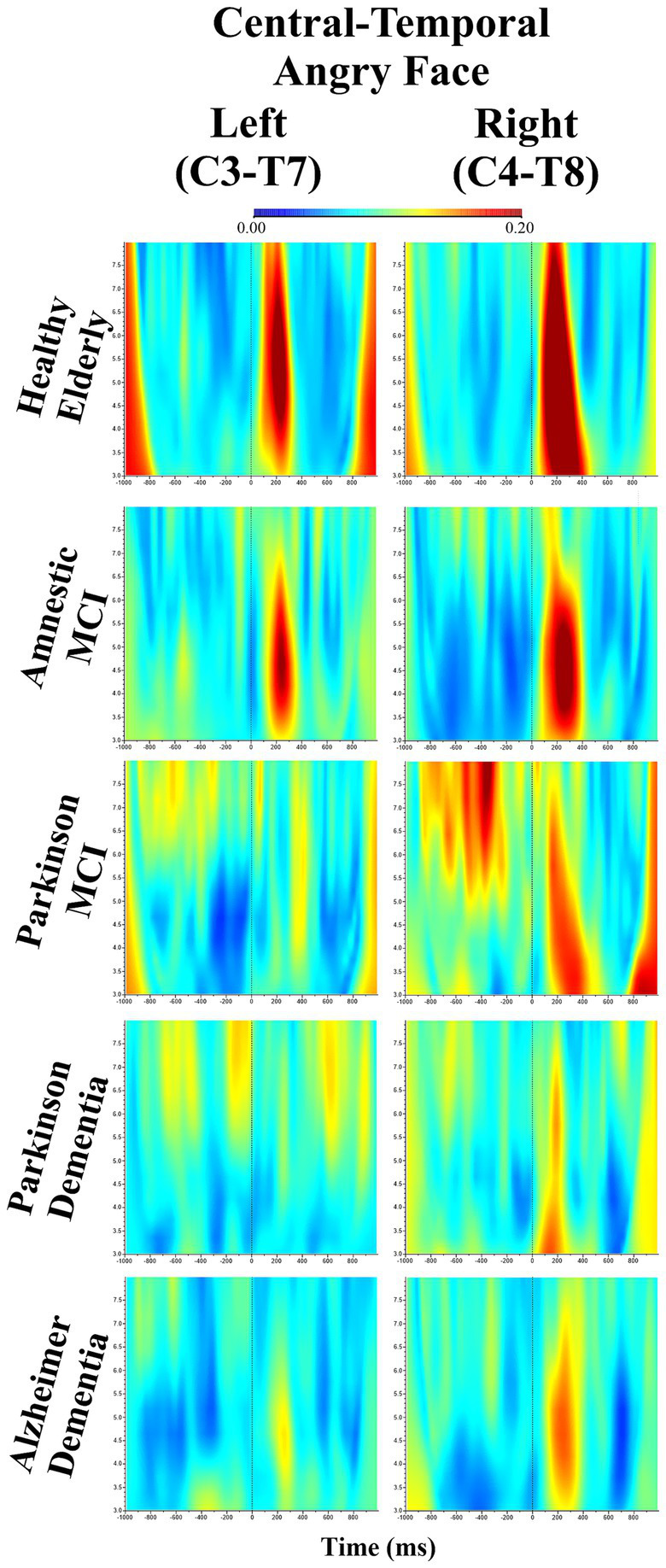
Topographic network graph displaying the inter-hemispheric event-related EEG theta spectral coherence values (200 ms) in all electrode pairs for each group (HE, aMCI, ADD, PD-MCI, and PDD) in the angry facial expression condition.

A third repeated measures ANOVA analysis was conducted to examine the differences in interhemispheric event-related EEG theta coherence values recorded from patient groups across different emotional facial expressions. This analysis had a 3 (Facial Expressions: angry, happy and neutral) X 6 (Locations: F3-F4 for frontal, C3-C4 for central, T7-T8 for temporal, TP7-TP8 for temporo-parietal, P3-P4 for parietal, and O1-O2 for occipital regions) X 5 (Groups: HE, aMCI, PD-MCI, ADD, PDD) design. A significant main effect of group was observed, lending initial support to the study hypothesis that abnormalities in event-related EEG theta coherence vary as a function of neurodegenerative disease type and severity (F (df = 4,101) = 8.122, *p* < 0.000, *ηp*^2^ = 0.243). To identify the specific group-level differences underlying this effect, *post hoc* comparisons were performed. The results revealed that PD-MCI (M = 0.0767, SE = 0.0211; *t*_101_ = 3.6293, *p* < 0.001, *p*_bonferroni_ = 0.004) and PDD groups (M = 0.0788, SE = 0.0207; *t*_101_ = 3.8043, *p* < 0.001, *p*_bonferroni_ = 0.002) had reduced inter-hemispheric event-related EEG theta spectral coherence values compared to HE group. Although the coherence values were also higher in the HE group relative to the ADD group this difference did not remain statistically significant after Bonferroni correction (M = 0.0516, SE = 0.0183; *t*_101_ = 2.8182, *p* = 0.006, *p*_bonferroni_ = 0.058). In addition to the differences relative to the HE group, PD-MCI (M = 0.0827, SE = 0.0211; *t*_101_ = 3.9120, *p* < 0.001, *p*_bonferroni_ = 0.002), ADD (M = 0.0575, SE = 0.0183; *t*_101_ = 3.1445, *p* = 0.002, *p*_bonferroni_ = 0.022), and PDD groups (M = 0.0848, SE = 0.0207; *t*_101_ = 4.0926, *p* < 0.001, *p*_bonferroni_ < 0.001) demonstrated significantly lower coherence values compared to aMCI. No significant differences were observed among the patient groups themselves. Taken together these findings suggest that the observed group differences did not appear to follow a clear disease-type-specific pattern. Instead, they may reflect the influence of disease severity, as only the aMCI group showed significantly higher event-related EEG theta coherence values compared to the other groups (PD-MCI, ADD, and PDD), whereas the HE group differed significantly only from the PD-MCI and PDD groups. These group differences are visually presented in Figure 4. Beyond this main effect, significant interactions were observed between facial expression and group (*F*(df = 8, 200) = 2.6530, *p* = 0.009, *ηp*^2^ = 0.0951), and between location and group (*F*(df = 13, 335) = 1.9407, *p* = 0.024, *ηp*^2^ = 0.0714). Moreover, a three-way interaction among facial expression, location, and group (*F*(df = 32, 820) = 1.4888, *p* = 0.040, *ηp*^2^ = 0.0557) was also significant. To further explore the nature of these interactions, post hoc comparisons were conducted. The results of comparisons regarding the facial expression and group interaction indicated that significant differences compared to the HE group were limited to the angry facial expression condition. However, for comparison purposes, group-level event-related theta coherence values across all facial expression conditions are shown in Figure 5, with time-frequency (Wavelet) visualizations of each facial expression across groups provided for the occipital electrode pair in Figure 6. Initially, the PD-MCI (M = 0.0944, SE = 0.0267; *t*_101_ = 3.5400, *p* < 0.001, *p*_bonferroni_ = 0.064), ADD (M = 0.0785, SE = 0.0231; *t*_101_ = 3.4003, *p* < 0.001, *p*_bonferroni_ = 0.101), and PDD (M = 0.0997, SE = 0.0261; *t*_101_ = 3.8156, *p* < 0.001, *p*_bonferroni_ = 0.025) groups all showed reduced interhemispheric theta coherence compared to the HE group. However, only the difference between the PDD and HE groups remained statistically significant following the correction, though this should be interpreted with caution given the conservative nature of the Bonferroni correction. Additionally, it is worth noting that in the angry facial expression condition PD-MCI (M = 0.1052, SE = 0.0267; *t*_101_ = 3.9460, *p* < 0.001, *p*_bonferroni_ = 0.015), ADD (M = 0.0893, SE = 0.0231; *t*_101_ = 3.8691, *p* < 0.001, *p*_bonferroni_ = 0.020) and PDD groups (M = 0.1105, SE = 0.0261; *t*_101_ = 4.2298, *p* < 0.001, *p*_bonferroni_ = 0.005) exhibited significantly reduced theta coherence compared to aMCI. This is also visually evident in the inter-hemispheric coherence differences illustrated in Figure 7, which displays topographic network graphs for each group in the angry facial expression condition. All comparison results are shown in Table 2. The comparisons regarding the location and group interaction revealed several initial differences in theta coherence between the HE and patient groups (ADD, PD-MCI, and PDD) across inter-hemispheric electrode pairs (*F*(df = 13, 335) = 1.9407, *p* = 0.024, *ηp*^2^ = 0.0714). While the ADD group showed reductions at temporal and temporoparietal sites, PD-MCI and PDD groups exhibited more widespread reductions. However, none of these differences remained significant after Bonferroni correction. These initial effects may suggest a more widespread disruption of inter-hemispheric coherence in PD patients, contrasted with a more region-specific (temporal) vulnerability in AD patients.

**Figure 8:**
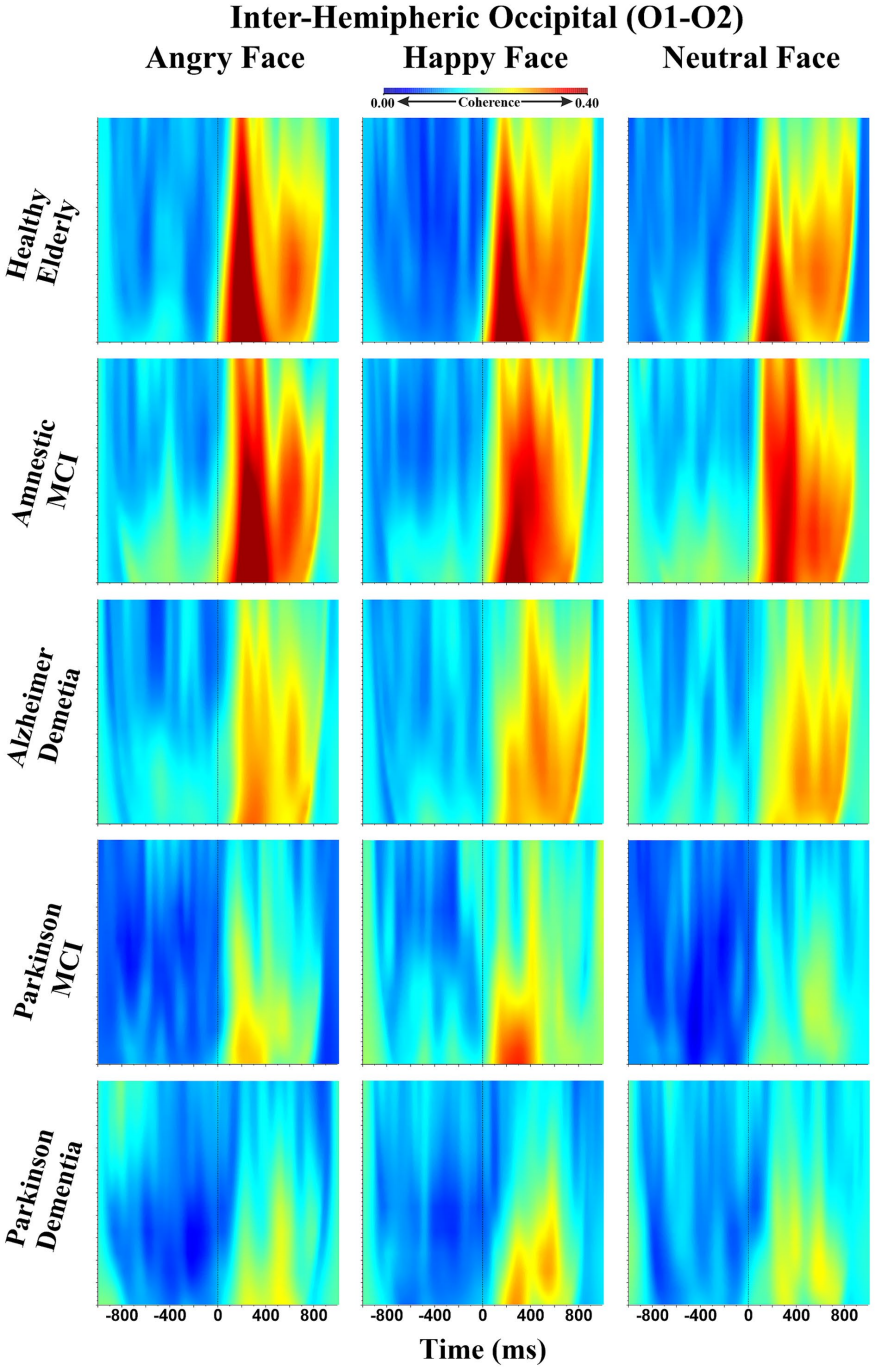
A line graph displaying group differences of the inter-hemispheric event-related EEG theta coherence values by location; red line shown with circle represents HE, blue line shown with square represents aMCI, green line shown with rhombus represents PD-MCI, pink line shown with triangle represents ADD, and black line shown with disk represents PDD.

There was a significant main effect of facial expression (*F*(df = 2,202) = 6.146, *p* = 0.003, *ηp*^2^ = 0.057). Further comparisons to examine the source of this difference revealed that event-related theta spectral coherence values elicited by angry facial expression stimuli were greater than those that are elicited by neutral (M = 0.0216, SE = 0.0065; *t*_101_ = 3.3418, *p* = 0.001, *p*_bonferroni_ = 0.004) and happy facial expression stimuli (M = 0.0130, SE = 0.0060; *t*_101_ = 2.1505, *p* = 0.034, *p*_bonferroni_ = 0.102). However, the difference between angry and happy facial expression conditions were no longer significant after correction. Finally, there was a significant main effect of location (*F*(df = 5,505) = 28.2014, *p* < 0.001, *ηp*^2^ = 0.2183), suggesting that inter-hemispheric event-related EEG theta spectral coherence values varied across electrode pairs. *Post hoc* comparisons carried out to further investigate this variation revealed that inter-hemispheric event-related theta coherence values were greater at occipital electrode pairs compared to frontal (M = −0.1382, SE = 0.0172; *t*_101_ = −8.0323, *p* < 0.001, *p*_bonferroni_ < 0.001), central (M = −0.1108, SE = 0.0174; *t*_101_ = −6.3574, *p* < 0.001, *p*_bonferroni_ < 0.001), and parietal pairs (M = −0.1289, SE = 0.0180; *t*_101_ = −7.1620, *p* < 0.001, *p*_bonferroni_ < 0.001); at temporoparietal compared to frontal (M = −0.1007, SE = 0.0154; *t*_101_ = −6.5236, *p* < 0.001, *p*_bonferroni_ < 0.001), central (M = −0.0732, SE = 0.0135; *t*_101_ = 5.4098, *p* < 0.001, *p*_bonferroni_ < 0.001), and parietal locations (M = 0.0914, SE = 0.0128; *t*_101_ = 7.1663, *p* < 0.001, *p*_bonferroni_ < 0.001); at temporal compared to frontal (M = −0.0808, SE = 0.0134; *t*_101_ = −6.0510, *p* < 0.001, *p*_bonferroni_ < 0.001), central (M = −0.0534, SE = 0.0126; *t*_101_ = −4.2261, *p* < 0.001, *p*_bonferroni_ < 0.001), and parietal sites (M = 0.0716, SE = 0.0135; *t*_101_ = 5.3087, *p* < 0.001, *p*_bonferroni_ < 0.001). Inter-hemispheric event-related EEG theta coherence did not significantly differ among occipital, temporoparietal, and temporal electrode pairs, or among frontal, central, and parietal electrode pairs. The distribution of coherence values across these electrode pairs for each group is illustrated in Figure 8.

The significant three-way interaction among facial expression, location, and group prompted separate repeated-measures ANOVAs for each facial expression to examine region- and group-specific differences in coherence (see Table 3 and Supplementary Tables for detailed statistical values). For the angry facial expression, significant main effects of location and group were observed, along with a location × group interaction; *post hoc* tests revealed reduced theta coherence in ADD (*p*_bonferroni_ = 0.010), PD-MCI (*p*_bonferroni_ = 0.006), and PDD (*p*_bonferroni_ = 0.002) groups compared to the HE group. Similarly, significant main effects and interactions were found for both happy and neutral facial expressions; specifically, the PDD group exhibited significantly lower coherence in response to happy faces HE > PDD (M = 0.0838, SE = 0.0239; *t*_101_ = 3.5076, *p* < 0.001, *p*_bonferroni_ = 0.007), while the PD-MCI group showed significantly reduced coherence relative to HE in the neutral condition HE > PD-MCI (M = 0.0727, SE = 0.0211; *t*_101_ = 3.4432, *p* < 0.001, *p*_bonferroni_ = 0.008). Although various region-specific differences were initially observed across all expressions, it is critical to note that none of the location × group interaction effects remained statistically significant following Bonferroni correction, suggesting that these initial uncorrected findings should be interpreted with caution.

**Table 3:** All comparisons and significance levels according to facial expressions.

Comparison	Angry face	Neutral face	Happy face
	Group comparisons and interaction details (post-hoc)		
Group***	HE > PD-MCI**, ADD*, PDD**, aMCI	HE > PD-MCI**	HE > PDD**
Location***	tp > f, c, p (All ***) t > f***, c*, p*** o > f, c, p (All ***)	tp > f, c, p (All ***) t > f, c, p (All ***) o > f, c, p (All ***)	tp > f, c, p (All ***) t > f***, c*, p** o > f***, c***, t*, p***
Group × Location*	HE > PD-MCI: t, tp, o HE > PDD: c, o HE > ADD: t, tp, o	HE > PD-MCI: c, t, tp, o HE > PDD: c, tp HE > ADD: tp	HE > PD-MCI: c, tp HE > PDD: c, tp, o HE > ADD: t, tp

(*p* OR *p*_bonferroni:_ *<0.05, **<0.01, ***<0.001), HE: Healthy Elderly, aMCI: Amnestic Mild Cognitive Impairment, PD-MCI: Parkinson’s Disease-Mild Cognitive Impairment, ADD: Alzheimer’s Disease Dementia, PDD: Parkinson’s Disease Dementia, f: frontal, c: central, t: temporal, tp: temporoparietal, p: parietal, o: occipital.

To sum up, significant main effects of both location and group were observed across all three repeated measures ANOVA models carried out for each facial expression condition. Event-related theta coherence values consistently varied by electrode site, with higher values at temporoparietal, temporal, and occipital locations compared to central, frontal, and parietal sites, with occipital sites exhibiting the highest values. Group differences were most pronounced in the angry facial expression condition, where PD-MCI, ADD, and PDD groups showed reduced coherence compared to HE. Event-related theta coherence was significantly reduced in the PDD group compared to HE during the happy condition, and in the PD-MCI group during the neutral condition. Although interactions between location and group were detected in each condition, post hoc comparisons did not survive Bonferroni correction. Nevertheless, a similar uncorrected pattern emerged across expressions: the PD-MCI and PDD groups tended to show reduced coherence values at central, temporal, temporoparietal, and occipital sites, whereas the ADD group showed reductions primarily at temporal and temporoparietal locations. Notably, in the angry facial expression condition, ADD group showed reduced coherence at the occipital electrode pair compared to the HE.

Finally, gender was included as a between-subjects factor in all six ANOVA models as an exploratory control. No significant main effects of Gender or Gender × Group interactions were observed in any of the analyses (all *p*-values > 0.05), suggesting that gender-related processing differences do not appear to account for the observed group differences in event-related EEG theta spectral coherence.

## 4 Discussion

This study aimed to investigate functional connectivity in response to emotional facial expressions across five groups reflecting healthy aging and varying levels of cognitive impairment: HE, aMCI, ADD, PD-MCI, and PDD, with particular attention to the role of the right hemisphere in emotional processing. Specifically, intra- and inter-hemispheric event-related EEG theta spectral coherence values were calculated from neural responses to angry, happy, and neutral facial expressions. The focus on theta-band activity was based on its association with limbic system engagement (Buzsáki, 2002) and its involvement in supporting long-range communication between brain regions (Von Stein and Sarnthein, 2000). Prior studies have shown that theta oscillations are highly involved in attention (Tan et al., 2024), memory (Herweg et al., 2020), and emotional processing (Knyazev et al., 2009), and tend to increase in response to emotionally salient stimuli relative to neutral ones (Aftanas et al., 2001). To begin with the comparison of intra-hemispheric event-related EEG theta spectral coherence, no significant group differences were observed relative to healthy controls. However, overall event-related EEG theta coherence was higher in the right hemisphere compared to the left, providing potential support to the proposed role of the right hemisphere in emotional processing. In contrast to the intra-hemispheric coherence findings, the inter-hemispheric event-related EEG theta coherence values exhibited significant group differences, with PD-MCI and PDD groups having reduced coherence compared to HE. These inter-hemispheric reductions may reflect structural disconnection affecting callosal white matter pathways that support inter-hemispheric information transfer. In Parkinson’s disease, cognitive impairment has been associated with corpus callosum white matter abnormalities (Bledsoe et al., 2018). In Alzheimer’s disease, multimodal MRI–EEG evidence has demonstrated correlations between corpus callosum integrity and inter-hemispheric EEG coherence (Pogarell et al., 2005; Teipel et al., 2009), suggesting that reduced theta coherence may represent the functional expression of disrupted callosal connectivity. Throughout this section, results are first evaluated separately with respect to intra- and inter-hemispheric event-related EEG theta spectral coherence to ensure clarity, although overlapping patterns are addressed later where relevant.

Beyond the primary findings outlined above, the analyses of intra-hemispheric event-related EEG theta spectral coherence values revealed additional effects. Although there was no significant overall group difference as previously reported, uncorrected comparisons indicated that the PD-MCI, ADD, and PDD groups showed reduced theta coherence at central-temporal electrode pairs, and that the PD-MCI and ADD groups also showed reductions at central-temporoparietal pairs. However, none of these remained significant after correction. Given the strict nature of the Bonferroni correction, the absence of significance should be interpreted with caution, as the observed reductions at central, temporal and temporoparietal sites may still be informative regarding the underlying group differences related to Alzheimer’s and Parkinson’s disease and should not be dismissed outright. Moreover, the significant main effects of Facial Expression, Hemisphere, and Location suggest that theta coherence values differed significantly in response to angry facial expressions compared to neutral ones, with higher coherence observed in the right hemisphere and at central-temporoparietal and central-occipital electrode connections. While the overall analysis of intra-hemispheric coherence revealed that the right hemisphere demonstrated higher values than the left, an additional ANOVA was conducted using hemisphere-averaged coherence values to determine whether this asymmetry varied as a function of Facial Expression or Group. This simpler model, which included Facial Expression, Hemisphere, and Group as factors, revealed no significant three-way interaction, suggesting that the observed right-hemispheric predominance in intra-hemispheric coherence—reflecting higher functional connectivity among electrode pairs within the right hemisphere—was consistent across emotional expressions and groups. Taken together, these findings align with previous evidence supporting higher right-hemisphere involvement in the processing of emotional facial expressions (Güntürkün et al., 2020; Kheirkhah et al., 2021) and with reports of greater impairment in recognizing negative emotional expressions in Parkinson’s (Argaud et al., 2018) and Alzheimer’s disease (Fernández-Ríos et al., 2021).

With respect to inter-hemispheric EEG theta coherence results, a significant group difference was observed, marked by reduced coherence in the PD-MCI and PDD groups compared to healthy controls. Further comparisons revealed that in the angry facial expression condition, the PDD group exhibited reduced coherence relative to HE, and the PD-MCI, ADD, and PDD groups showed lower values relative to the aMCI group. Findings also revealed initially significant differences across recording sites in the PD-MCI, ADD, and PDD groups compared to the HE. Specifically, coherence reductions appeared more widespread in the PD-MCI and PDD groups, affecting central, temporoparietal, and occipital sites, whereas the ADD group showed reductions primarily at temporal sites. This spatial pattern is consistent with functional neuroimaging evidence demonstrating early involvement of large-scale posterior association networks in Alzheimer’s disease (Dickerson and Sperling, 2008). Moreover, fMRI work in mild AD has shown altered responsivity within temporal–limbic components of the face-emotion processing network, including amygdala and temporal lobe regions (Wright et al., 2007), providing converging evidence that emotion-related network integration may be disrupted in AD. Although these effects did not remain significant after correction, they suggested a pattern of impairment potentially relevant to Alzheimer’s and Parkinson’s disease, in line with the literature (Dickerson and Sperling, 2008; Suo et al., 2022; Zawiślak-Fornagiel et al., 2023). In Parkinson’s disease, resting-state fMRI studies have demonstrated altered cortical and subcortical connectivity patterns associated with cognitive impairment (Yeager et al., 2024), supporting a network-level interpretation of the present coherence reductions. While steps were taken to mitigate the potential effects of volume conduction during EEG signal analysis in the current study, the results should be interpreted with caution due to the inherent spatial resolution limitations of EEG, which restrict precise localization of neural sources underlying the observed coherence patterns. Inter-hemispheric event-related EEG theta spectral coherence values in the angry facial expression condition were found to be significantly higher than those in the neutral condition, and greater at occipital, temporal, and temporoparietal electrode sites relative to frontal, central, and parietal locations, indicating higher communication in these electrode pairs. Moreover, when examined individually within condition-specific ANOVAs, each facial expression condition revealed a consistent spatial pattern, with inter-hemispheric event-related EEG theta coherence values at temporoparietal, temporal, and occipital electrode pairs significantly higher than those observed at frontal, central, and parietal sites. Within these models, group comparisons showed that in the angry facial expression condition, the PD-MCI, ADD, and PDD groups exhibited reduced event-related theta coherence, whereas in the happy and neutral conditions, only the PDD and PD-MCI groups, respectively, showed lower coherence values. Importantly, the fact that these reductions were predominantly observed in the angry facial expression condition lends further support to the vulnerability in recognizing negative emotional expressions associated with both PD (Kan et al., 2002; Yuvaraj et al., 2015) and AD (Güntekin et al., 2019).

When considered together, intra- and inter-hemispheric event-related EEG theta spectral coherence values were consistently higher in response to angry facial expressions compared to neutral ones, in line with previous evidence that unpleasant and emotionally negative facial expressions elicit higher EEG theta coherence than neutral or positive facial expressions in healthy adults (Kostandov and Cheremushkin, 2011). Given that spectral coherence values theoretically range from 0 (indicating no functional connection) to 1 (indicating perfect synchronization), these increases reflect enhanced functional communication in response to emotionally salient (specifically negative) stimuli, as happy facial expressions did not elicit significant coherence elevations in either intra- or inter-hemispheric connections. Notably, these increases were most pronounced within the right hemisphere and across inter-hemispheric temporal, temporoparietal, and occipital electrode pairs. Also, PD-MCI, ADD and PDD groups showed significantly reduced inter-hemispheric EEG theta coherence at these electrode pairs (temporal, temporoparietal, and occipital), possibly indicating functional disruption in areas that are highly involved in emotional processing (Petro et al., 2025).

High-level association areas in the temporal, parietal, and frontal regions, critical for nearly all cognitive functions (Stam, 2014), are among the most affected regions in AD (Menardi et al., 2018). In addition, multimodal MRI–EEG evidence supports a structural–functional coupling between callosal integrity and inter-hemispheric EEG coherence in Alzheimer’s disease, with region-specific associations between corpus callosum size and inter-hemispheric coherence (Pogarell et al., 2005). Related work combining EEG coherence and diffusion MRI further links inter-hemispheric coherence patterns to the integrity of underlying inter-hemispheric fiber tracts in aging and amnestic MCI (Teipel et al., 2009). Notably, the hippocampus and amygdala, which are among the key regions in generating theta oscillations (Pignatelli et al., 2012), are typically disrupted in AD (Punzi et al., 2024) and have been consistently implicated in emotional processing (Palomero-Gallagher and Amunts, 2022). While the EEG method employed in this study does not allow for precise neuroanatomical localization, the observed reductions in theta coherence over temporal and posterior electrode sites may correspond to disruptions in underlying regions frequently implicated in emotional facial expression processing. Furthermore, dopamine cycle disruption in structures such as the basal ganglia, insula, and amygdala in PD is suggested to affect the processing of emotional facial expressions such as angry, scared, and disgusted (Calder et al., 2001; Suzuki et al., 2006; Ariatti et al., 2008). Moreover, observations of impaired inter-hemispheric EEG theta coherence responses (Yuvaraj et al., 2015) and EEG theta relative power (Yuvaraj et al., 2013) during emotional facial expression recognition in PD highlights the suitability of theta oscillations as a physiological correlate of emotional processing deficits, further supporting their use in comparing the pathological patterns of PD and AD. Although EEG coherence alone cannot directly confirm regional impairment, the spatial distribution of these effects aligns with known patterns of functional disruption in AD and PD, offering a plausible link between altered event-related EEG theta spectral coherence and emotion-related deficits.

Subjects in the neurocognitive disorder groups in our sample were expected to exhibit reduced coherence compared to healthy controls; however, this pattern was not observed in the aMCI group. In fact, in some analyses, aMCI participants showed higher coherence values than HE, although these differences did not reach statistical significance. Notably, the aMCI group demonstrated significantly higher inter-hemispheric EEG theta spectral coherence than the PD-MCI, ADD, and PDD groups, a pattern that was also evident in the angry facial expression condition as observed in the *post hoc* comparisons (Facial Expression X Group). These findings could be explained by compensatory mechanisms that activate neuroplasticity-related circuits in the earlier stages of the disease (Bajo et al., 2010; Bonanni et al., 2021), as previously suggested in a study comparing EEG event-related theta coherence in aMCI and AD patients during a visual oddball paradigm, where higher values in the aMCI group relative to AD and healthy controls were interpreted in this context (Fide et al., 2023). Converging multimodal evidence also suggests that early-stage network alterations may involve stage-dependent reconfiguration of inter-hemispheric coupling, where EEG-derived inter-hemispheric coherence relates to the integrity of underlying inter-hemispheric fiber systems in aging and amnestic MCI (Teipel et al., 2009), supporting the plausibility of compensatory network recruitment prior to more overt disconnection. Similarly, a comparable trend was reported by Jiang and Zheng (2006), where higher connectivity values were observed in MCI patients compared to healthy controls during a working memory task. This difference was specifically observed in the frontal, central, parietal, and temporal regions. Given that working memory-related processes require stronger communication among the lateral prefrontal cortex, posterior association, and temporal areas, these findings could be interpreted as pathology-related changes in these processes. This increase has been argued to reflect an adaptive response to neuropathology (Hillary and Grafman, 2017), potentially linked to neural compensation mechanisms associated with individuals’ cognitive reserve levels, which are influenced by various lifestyle factors (Barulli and Stern, 2013). Overall, these factors may contribute to altered network dynamics in aMCI, manifested as increased inter-hemispheric connectivity.

All in all, the current study had limitations. An important limitation is the absence of a Parkinson’s disease group without cognitive impairment. Although inclusion of such a group would have allowed clearer separation of Parkinson’s disease–specific effects from those related to cognitive decline, the present design focused on clinically manifest MCI and dementia stages. In clinical populations, identifying Parkinson’s patients with preserved cognition who meet strict neuropsychological criteria can be challenging, particularly in older samples where subtle cognitive changes are common. Therefore, the findings should be interpreted as reflecting disease type and cognitive severity rather than the isolated effect of Parkinson’s pathology. Moreover, PD-MCI and PDD groups differ from AD and healthy elderly participants in motor symptom severity and dopaminergic treatment, which may influence oscillatory dynamics; thus, medication- and motor-related factors cannot be entirely excluded when interpreting the observed group differences.

Despite the relatively large sample size, imbalances in group sizes and gender distribution may have limited the generalizability of the findings. Given the gender imbalance in the sample and the exclusive use of female facial expression stimuli, gender was included as a between-subjects factor in all six ANOVA models to control for potential gender-related effects. Although no significant Gender or Gender × Group effects were found, a potential confounding influence of gender cannot be entirely ruled out. Thus, accounting for gender balance in future research remains important to enhance the interpretability of group-level effects. A further limitation concerns the neuropsychological and emotional assessments administered for diagnostic purposes. Although these measures informed group classification, they were not included as covariates in the EEG analyses. Therefore, it cannot be entirely excluded that some of the observed coherence differences may partly reflect inter-group differences in cognitive performance rather than disease category alone.

In addition, coherence analysis tends to exhibit higher variability and greater sensitivity to artifacts (e.g., volume conduction) compared to other functional connectivity methods. However, to enhance the accuracy the calculations, several preventive measures were implemented, including the use of a Laplacian filter, Wavelet Transform, Fisher’s Z transformation, and an increased number of stimulus trials. Future research could replicate this study design with a larger sample size that has a more balanced sample. Increasing the number of electrodes and incorporating longitudinal follow-up would also contribute to a more precise understanding of connectivity changes across disease progression.

Taken together, the present study examined the intra- and inter-hemispheric event-related EEG theta spectral coherence in response to emotional facial expressions across five groups: aMCI, PD-MCI, ADD, PDD, and HE. The intra-hemispheric findings revealed that event-related EEG theta spectral coherence values did not significantly differ across groups, thus not supporting the initial hypothesis regarding group-level differences. However, coherence was significantly higher in response to angry facial expressions compared to neutral ones, particularly in the right hemisphere and at central-temporoparietal and central-occipital electrode pairs. On the other hand, inter-hemispheric event-related EEG theta coherence was significantly reduced in the PD-MCI and PDD groups, with the PDD group showing this reduction particularly in response to angry facial expressions. Coherence values were also greater at occipital, temporal, and temporoparietal electrode sites relative to frontal, central, and parietal sites. Notably, the aMCI group exhibited significantly higher coherence than PD-MCI, ADD, and PDD groups in angry facial expression condition, pointing to potential compensatory brain mechanisms in the earlier stages of cognitive decline. Taken together, the intra- and inter-hemispheric coherence findings highlight significant disruptions specifically in response to angry facial expressions, supporting the view that the recognition of negative emotions is particularly vulnerable to pathological changes in AD and PD. These reductions were primarily observed at temporal, temporoparietal and occipital electrode pairs, regions commonly implicated in emotional processing. Although the findings partly supported the initial hypothesis by demonstrating significant event-related EEG theta spectral coherence reductions particularly in the dementia stage, gender imbalance across the groups and spatial resolution limitations of the EEG method should be considered when interpreting the results. Future studies could address these limitations by including larger, more gender-balanced samples and incorporating multimodal imaging methods to better localize the neural sources of the observed effects. Overall, this study provides valuable insights into how pathological changes in AD and PD affect spectral coherence responses to emotional facial expressions, and how these effects vary across different stages of the diseases.

## Data Availability

The raw data supporting the conclusions of this article will be made available by the authors, without undue reservation.
