# Supplementary material for: Impaired event-related theta spectral coherence in emotional facial expression processing in neurodegenerative disorders
Source: Front Hum Neurosci. 2026 Apr 16;20:1708832. doi: 10.3389/fnhum.2026.1708832 (PMC13128654; doi:10.3389/fnhum.2026.1708832)
Supplement: Supplementary file 1 [file Table_1.docx]

# Supplementary Material

## Inter-hemispheric

**Table 1: Theta Coherence Comparisons Between Groups (lower than HE Group)**

| **Group** | **Location** | **M (SE)** | **t_101_** | ***p*** | **​*p*_bonferroni_** |
| --- | --- | --- | --- | --- | --- |
| ADD | Temporal | 0.0945 (0.0357) | 2.6510 | .009 | 1.0 |
|  | Temporoparietal | 0.1147 (0.0375) | 3.0618 | .003 | 1.0 |
| PD-MCI | Central | 0.0744 (0.0281) | 2.6460 | .009 | 1.0 |
|  | Temporal | 0.0985 (0.0412) | 2.3924 | .019 | 1.0 |
|  | Temporoparietal | 0.1390 (0.0433) | 3.2143 | .002 | .764 |
|  | Occipital | 0.1289 (0.0548) | 2.3521 | .021 | 1.0 |
| PDD | Central | 0.0813 (0.0276) | 2.9500 | .004 | 1.0 |
|  | Temporoparietal | 0.1406 (0.0424) | 3.3157 | .001 | .553 |
|  | Occipital | 0.1595 (0.0537) | 2.9692 | .004 | 1.0 |

M: Mean, SE: Standard Error, t: t-value with degrees of freedom, p: Probability value, *p*_bonferroni_**_:_** Bonferroni corrected p-value

Note: Although all these values ​​detailed in the table appear significant relative to nominal *p* values, none of the differences remained statistically significant after applying the Bonferroni correction.

**Table 2: ANOVA Main Effects and Interactions**

| **Face** | **Effect/Interaction** | **Statistical Values** | ***p*** | **ηp^2^** |
| --- | --- | --- | --- | --- |
| Angry | Location | F(4, 383) = 22.5375 | <.001 | 0.1824 |
|  | Group | F(4, 101) = 9.0433 | <.001 | 0.2637 |
|  | Location x Group | F(15, 383) = 1.7715 | .036 | 0.0656 |
| Happy | Location | F(3, 353) = 24.5045 | <.001 | 0.1952 |
|  | Group | F(4, 101) = 5.4801 | <.001 | 0.1783 |
|  | Location x Group | F(14, 353) = 1.7416 | .046 | 0.0645 |
| Neutral | Location | F(3, 349) = 19.7258 | <.001 | 0.1634 |
|  | Group | F(4, 101) = 5.1269 | <.001 | 0.1688 |
|  | Location x Group | F(14, 349) = 2.0412 | .015 | 0.0748 |

M: Mean, SE: Standard Error, t: t-value with degrees of freedom, p: Probability value, *p*_bonferroni_**_:_** Bonferroni corrected p-value

**Table 3: Post-hoc Group Comparisons (lower than HE Group)**

| **Face** | **Group** | **M** | **t_101_** | ***p*** | **​*p*_bonferroni_** | **M (SE)** |
| --- | --- | --- | --- | --- | --- | --- |
| Angry | PD-MCI | 0.0944 | 0.0267 | 3.5400 | <.001 | .006 |
|  | ADD | 0.0785 | 0.0231 | 3.4003 | <.001 | .010 |
|  | PDD | 0.0997 | 0.0261 | 3.8156 | <.001 | .002 |
|  | aMCI | -0.0108 | 0.0231 | -0.4688 | .640 | 1.0 |
| Happy | PDD | 0.0838 | 0.0239 | 3.5076 | <.001 | .007 |
| Neutral | PD-MCI | 0.0727 | 0.0211 | 3.4432 | <.001 | .008 |

M: Mean, SE: Standard Error, t: t-value with degrees of freedom, p: Probability value, *p*_bonferroni:_ Bonferroni corrected p-value

**Table 4: Location x Group Interaction (lower than HE Group)**

| **Face** | **Group** | **Location** | **M** | **SE** | **t_101_** | ***p*** | ***p*_bonferroni_** |
| --- | --- | --- | --- | --- | --- | --- | --- |
| Angry | PD-MCI | Temporal | 0.1194 | 0.0553 | 2.1570 | .033 | 1.0 |
|  |  | Temporoparietal | 0.1717 | 0.0510 | 3.3673 | .001 | .468 |
|  |  | Occipital | 0.1522 | 0.0605 | 2.5152 | .013 | 1.0 |
|  | PDD | Central | 0.0982 | 0.0370 | 2.6551 | .009 | 1.0 |
|  |  | Occipital | 0.2079 | 0.0593 | 3.5041 | <.001 | .298 |
|  | ADD | Temporal | 0.1361 | 0.0479 | 2.8395 | .005 | 1.0 |
|  |  | Temporoparietal | 0.1343 | 0.0442 | 3.0424 | .003 | 1.0 |
|  |  | Occipital | 0.1071 | 0.0524 | 2.0443 | .044 | 1.0 |
| Happy | PD-MCI | Central | 0.0917 | 0.0299 | 3.0685 | .003 | 1.0 |
|  |  | Temporoparietal | 0.1211 | 0.0480 | 2.5201 | .013 | 1.0 |
|  | PDD | Central | 0.0890 | 0.0293 | 3.0379 | .003 | 1.0 |
|  |  | Temporoparietal | 0.1841 | 0.0471 | 3.9091 | <.001 | .073 |
|  |  | Occipital | 0.1727 | 0.0622 | 2.7782 | .007 | 1.0 |
|  | ADD | Temporal | 0.0802 | 0.0370 | 2.1682 | .032 | 1.0 |
|  |  | Temporoparietal | 0.1242 | 0.0416 | 2.9859 | .004 | 1.0 |
| Neutral | PD-MCI | Central | 0.0620 | 0.0266 | 2.3328 | .022 | 1.0 |
|  |  | Temporal | 0.0912 | 0.0455 | 2.0049 | .048 | 1.0 |
|  |  | Temporoparietal | 0.1244 | 0.0448 | 2.7777 | .007 | 1.0 |
|  |  | Occipital | 0.1303 | 0.0552 | 2.3586 | .020 | 1.0 |
|  | PDD | Central | 0.0567 | 0.0260 | 2.1773 | .032 | 1.0 |
|  |  | Temporoparietal | 0.1065 | 0.0439 | 2.4270 | .017 | 1.0 |
|  | ADD | Temporoparietal | 0.0855 | 0.0388 | 2.2060 | .030 | 1.0 |

M: Mean, SE: Standard Error, t: t-value with degrees of freedom, p: Probability value, ***p*_bonferroni:_** Bonferroni corrected p-value

Note: These differences did not remain significant after Bonferroni correction.

**Table 5: Location Main Effect Comparisons for Angry Facial Expression**

| **Comparison (Higher > Lower)** | **Degrees of freedom** | ***p*** | ***p*_bonferroni_** |
| --- | --- | --- | --- |
| Temporoparietal > Frontal, Central, Parietal | t = -5.64 / -4.34 / 6.67 | <.001 | <.001 |
| Temporal > Frontal, Central, Parietal | t = -5.72 / -3.28 / 4.93 | ≤.001 | ≤.021 |
| Occipital > Frontal, Central, Parietal | t = -7.54 / -5.42 / -6.77 | <.001 | <.001 |

M: Mean, SE: Standard Error, t: t-value with degrees of freedom, p: Probability value, *p*_bonferroni:_ Bonferroni corrected p-value

**Table 6. Summary of all comparisons and significance levels of the ANOVA analyses**

| **Analysis Type** | **ANOVA Main Effects & Statistical Values (F, p, ηp²)** | **Group Comparisons and Interaction Details (Post-hoc)** |
| --- | --- | --- |
| **Intra-hemispheric** | 3 Face x 7 Loc x 2 Hem x 5 Group |  |
| Group | (F (df = 4, 101) = 2.027, p = .096, ηp2 = .074) | - |
| Group x Location | (F (df = 10, 101) = 1.932, p = .040, ηp2 = .071) | central–temporal (C3-T7, C4-T8): HE > PD-MCI, ADD, PDD  central–temporoparietal (C3-TP7, C4-TP8): HE> PD-MCI, ADD  frontal–occipital locations (F3–O1, F4–O2): aMCI > PD-MCI, PDD |
| Face | (F (df =2,202) = 3.322, p = .038, ηp2 = 0.032)* | Angry > Neutral (M = 0.0097, SE = 0.0038; t101 = 2.5309, p = .013, *p*_bonferroni_ = .039)* |
| Hemisphere | (F (df =1,101) = 13.521, p = .000, ηp2 = 0.118)*** | Right > Left (M = -0.0140, SE = 0.0038; t101 = -3.6771, p < .001, *p*_bonferroni_ < .001)*** |
| Location | (F (df =6,606) = 9.287, p = .000, ηp2= 0.084)*** | *frontal-parietal < central-temporoparietal, central-occipital  *frontal-temporoparietal > frontal-parietal, frontal-occipital  ***Frontal–occipital < frontal-temporal, central–temporal, central–temporoparietal, central–occipital |
| **Hemispheric Asymmetry** | 3 Face x 2 Hem x 5 Group | ANOVA Main Effects: (F (df = 7, 191) = 1.0646, p = .389, ηp2 = 0.0405) |
| **Inter-hemispheric** | 3 Face x 6 Loc x 5 Group |  |
| Group | (F (df = 4,101) = 8.122, p < .000, ηp2= 0.243)*** | HE > PD-MCI**, PDD**  HE > ADD (p = .006, *p*_bonferroni_ = .058)  aMCI > PD-MCI (p <.001 , *p*_bonferroni_ = .002)**, ADD (p = .002, *p*_bonferroni_ = .022)*, PDD*** |
| Location | (F(df = 5,505) = 28.201, p <.001, ηp2 = 0.218)*** | ***occipital > frontal, central, and parietal  ***temporoparietal > frontal, central, parietal  ***Temporal > frontal, central, parietal  Inter-hemispheric event-related EEG theta coherence did not significantly differ among occipital, temporoparietal, and temporal electrode pairs, or among frontal, central, and parietal electrode pairs. |
| Group x Location | (F (df = 13, 335) = 1.9407, p = .024, ηp2 = 0.0714)* | HE >ADD: temporal, temporoparietal  HE > PD-MCI: central, temporal, temporoparietal, occipital  HE> PDD central, temporoparietal, occipital locations |
| Face | (F (df = 2,202) = 6.146, p = .003, ηp2= 0.057)** | Angry > neutral (p = .001, *p*_bonferroni_ = .004)**, happy (p = .034, *p*_bonferroni_ = .102) |
| Group x Face | (F (df = 8, 200) = 2.653, p = .009, ηp2 = 0.095)** | Angry: HE > PD-MCI (p <.001 , *p*_bonferroni_ = .064), ADD, PDD (p <.001 , *p*_bonferroni_ = .025)*  Angry: aMCI > PD-MCI (p <.001, *p*_bonferroni_ = .015)*, ADD (p <.001, *p*_bonferroni_ = .020)*, PDD** |
| Group x Face x Location | (F (df = 32, 820) = 1.488, p = .040 , ηp2 = 0.0557)*** | Based on this result, separate ANOVA comparisons were made for each facial expression in order to taking into account the sample, number of data and group size, to be explanatory. |
| **Angry Face** | 6 Loc x 5 Group |  |
| Group | (F (df = 4, 101) = 9.0433, p <.001, ηp2= 0.2637)*** | HE > PD-MCI**, ADD*, PDD**, aMCI (p = .640, *p*_bonferroni_ = 1.000) |
| Location | (F (df = 4, 383) = 22.5375, p <.001, ηp2= 0.1824)*** | ***Temporoparietal > frontal, central, parietal  *Temporal > frontal***, central (p = .001, *p*_bonferroni_ = .021)*, parietal***  ***Occipital > frontal, central, parietal |
| Group x Location | (F (df = 15, 383) = 1.7715, p = .036, ηp2= 0.0656)* | HE > PD-MCI: temporal, temporoparietal, occipital  HE > PDD: central, occipital  HE > ADD: temporal, temporoparietal, occipital |
| **Neutral Face** | 6 Loc x 5 Group |  |
| Group | (F(df = 4, 101) = 5.1269, p <.001, ηp² = 0.1688)*** | HE > PD-MCI (M = 0.0727, SE = 0.0211; t101 = 3.4432, p <.001, *p*_bonferroni_ = .008)** |
| Location | (F(df = 3, 349) = 19.7258, p <.001, ηp² = 0.1634)*** | ***Temporoparietal > frontal, central, parietal  ***Temporal > frontal, central, parietal  ***Occipital > frontal, central, parietal |
| Group x Location | (F(df = 14, 349) = 2.0412, p = .015, ηp² = 0.0748)* | HE > PD-MCI: central, temporal, temporoparietal, occipital  HE > PDD: central, temporoparietal  HE > ADD: temporoparietal |
| **Happy Face** | 6 Loc x 5 Group |  |
| Group | (F(df = 4, 101) = 5.4801, p <.001, ηp² = 0.1783)*** | HE > PDD (M = 0.0838, SE = 0.0239; t101 = 3.5076, p <.001, *p*_bonferroni_ = .007)** |
| Location | (F(df = 3, 353) = 24.5045, p <.001, ηp² = 0.1952)*** | ***Temporoparietal > frontal, central, parietal  *Temporal > frontal***, central (p = .002, *p*_bonferroni_ = .025)*, parietal (p <.001, *p*_bonferroni_ = .002)**  ***Occipital > frontal***, central***, temporal (p <.001, *p*_bonferroni_ = .012)*, parietal*** |
| Group x Location | (F(df = 14, 353) = 1.7416, p = .046, ηp² = 0.0645)* | HE > PD-MCI: central, temporoparietal  HE > PDD: central, temporoparietal, occipital  HE > ADD: temporal, temporoparietal |
| **Gender** | It was included as a between-subjects factor in all six ANOVA models as an exploratory control. | No significant main effects of Gender or Gender × Group interactions were observed in any of the analyses (all p-values >.05). |

(*p OR p*_bonferroni:_ **< .05,* ***< .01,* ****< .001),* M: Mean, SE: Standard Error, t: t-value with degrees of freedom, p: Probability value, *p*_bonferroni:_ Bonferroni corrected p-value*, HE: Healthy Elderly, aMCI: Amnestic Mild Cognitive Impairment, PD-MCI: Parkinson’s Disease-Mild Cognitive Impairment, ADD: Alzheimer’s Disease Dementia, PDD: Parkinson’s Disease Dementia.*
